# Mass diffusion coefficient measurement for vitreous humor using FEM and MRI

**DOI:** 10.1088/1757-899X/297/1/012024

**Published:** 2018

**Authors:** Komsan Rattanakijsuntorn, Anita Penkova, Satwindar S. Sadhal

**Affiliations:** 1 Faculty of Engineering, Department of Mechanical Engineering, Ubon Ratchathani University, 85 Sathonlamark Road, Warinchamrap, Ubon Ratchathani 34190, Thailand; 2 Aerospace & Mechanical Engineering Department, University of Southern California, Los Angeles, CA 90089-1453, United States

## Abstract

In early studies, the ‘contour method’ for determining the diffusion coefficient of the vitreous humor was developed. This technique relied on careful injection of an MRI contrast agent (surrogate drug) into the vitreous humor of fresh bovine eyes, and tracking the contours of the contrast agent in time. In addition, an analytical solution was developed for the theoretical contours built on point source model for the injected surrogate drug. The match between theoretical and experimental contours as a least square fit, while floating the diffusion coefficient, led to the value of the diffusion coefficient. This method had its limitation that the initial injection of the surrogate had to be spherical or ellipsoidal because of the analytical result based on the point-source model. With a new finite element model for the analysis in this study, the technique is much less restrictive and handles irregular shapes of the initial bolus. The fresh bovine eyes were used for drug diffusion study in the vitreous and three contrast agents of different molecular masses: gadolinium-diethylenetriaminepentaacetic acid (Gd-DTPA, 938 Da), non-ionic gadoteridol (Prohance, 559 Da), and bovine albumin conjugated with gadolinium (Galbumin, 74 kDa) were used as drug surrogates to visualize the diffusion process by MRI. The 3D finite element model was developed to determine the diffusion coefficients of these surrogates with the images from MRI. This method can be used for other types of bioporous media provided the concentration profile can be visualized (by methods such as MRI or fluorescence).

## 1. Introduction

During the past decades, many researchers have attempted to develop ocular drug delivery systems. Intravitreal drug delivery has become a popular method of treatment of many retinal diseases, commonly including AMD, Diabetic Retinopathy, and Retinal Vein Occlusions. While various drugs have been successfully developed, delivering drugs to the retina requires knowledge of the relevant fluid mechanics and transport phenomena. Low drug concentrations are insufficient to treat the retinal disease and high concentrations can carry risks of side effects [1–3]. Therefore, it is crucial to know the drug distribution within the eye following delivery by intravitreal injection.

With the help from the advanced computer software nowadays, ocular drug transport models have been developed to simulate and predict drug distribution in the eye [4–7]. These models require the values of parameters that are used in the governing equations, and one of the most common uses of parameter is drug diffusivity. Therefore, the aim of this work is to develop a technique that delivers the values of diffusion coefficient of drugs in the vitreous from MRI images.

In early study by Penkova et al [8], the ‘contour method’ for determining the diffusion coefficient of the vitreous humor was developed. This technique relied on careful injection of an MRI contrast agent (surrogate drug) into the vitreous humor of fresh bovine eyes, and tracking the contours of the contrast agent in time. In addition, an analytical solution was developed for the theoretical contours built on point source model for the injected surrogate drug. The match between theoretical and experimental contours as a least square fit, while floating the diffusion coefficient, led to the value of the diffusion coefficient. This method had its limitation that the initial injection of the surrogate had to be spherical or ellipsoidal because of the analytical result based on the point-source model. With a new finite element model for the analysis in this study, the technique is much less restrictive and handles irregular shapes of the initial bolus.

## 2. Methods and materials

### 2.1. Apparatus

Whole bovine eyes were prepared for MRI visualization with the same method as described in the previous study [8]. Three gadolinium-based contrast agents: gadolinium-diethylenetriamine pentaacetic acid (Gd-DTPA, 938 Da), nonionic gadoteridol (Prohance, 559 Da), and bovine albumin conjugated with gadolinium (Galbumin, 74 kDa) were used as drug surrogates to model diffusive transport of different molecular weight drugs. Experiments were conducted by injecting 30 μL of the contrast agents in the vitreous of the whole eye. With the concentration measurement from the MRI signal at regular intervals for 2–3 hours, the concentration contours were constructed and matched with the theoretical contours from the finite element analysis. The best fit at different times gave fairly consistent values of the diffusion coefficient.

### 2.2. Governing equations and model development

A three-dimensional finite element model of the vitreous was developed to help in analyzing MRI images and calculate diffusion coefficients by comparing simulated data to MRI experimental data. To model the drug distribution in the vitreous, the mass transport equation was applied:

(1) $$\frac{\partial c}{\partial t}+v\cdot \nabla c-D\nabla^{2}c+q=0$$

where c represents the concentration of the drug, D is the diffusion coefficient of the drug in the vitreous, v is the velocity of fluid in the vitreous, and q is the release rate of the drug. Since the experiment was conducted by excluding convection in the system, and we assumed that the drug is neither generated nor degraded within the vitreous, the terms involving with v and q were set to zero. The mass transport equation can be rewritten as:

(2) $$\frac{\partial c}{\partial t}-D\nabla^{2}c=0$$

**Figure 1:**
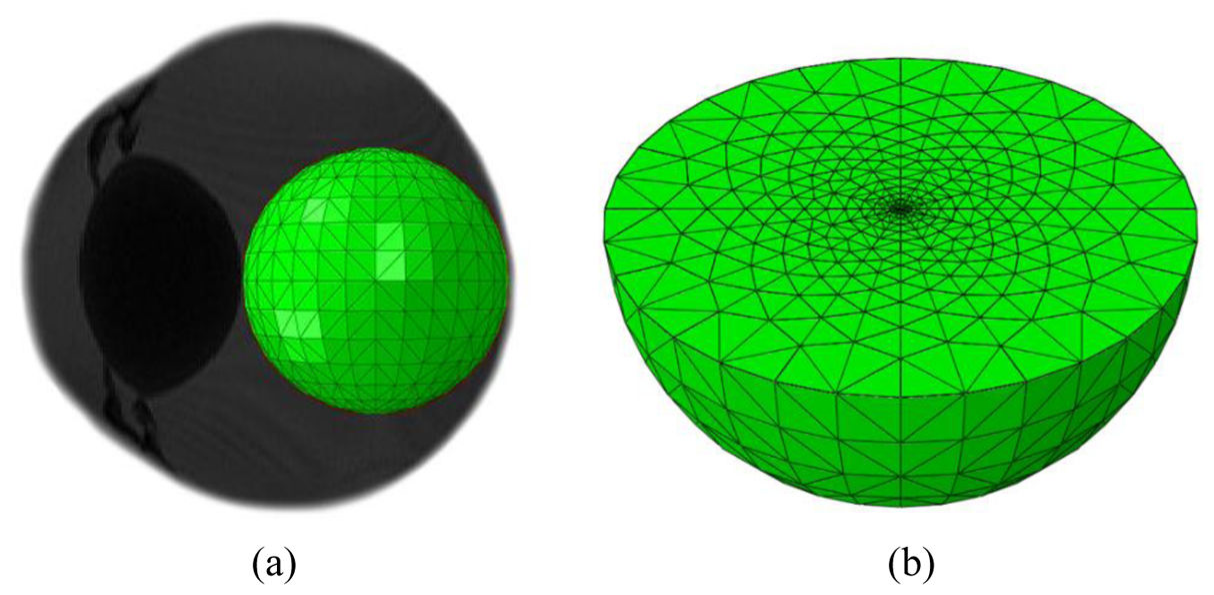
The domain of interest and the finite element tetrahedral meshes.

For the purpose of modelling, the domain of interest (of the vitreous) in which drugs diffused through was simply defined as a sphere with a radius of 10 mm as illustrated in figure 1(a). The geometry created for the domain of interest, were converted into a total of 105,276 finite element tetrahedral meshes. Since the rate of change of concentration over time near the center of the domain is larger than the outer, mesh size was varied by having finer meshes near the center and coarser meshes near the outer boundary as shown in figure 1(b).

### 2.3. Boundary and initial conditions

The vitreous humor in this study is considered to be a homogeneous substance. The lens is avascular and is considered to be impermeable to fluid flow, so we assumed that the drug surrogate does not penetrate the lens. Similarly, RPE is considered as a major barrier for the retinal delivery of hydrophilic drugs, the same assumption that we made for the lens also applied for the RPE. Since the outer boundary of the domain of interest reaches the regions of lens and RPE membrane, we simplified the boundary condition at the outer surface as zero flux condition. This kind of boundary condition would much simplify the finite element equation by eliminating the flux term as detailed in appendix A. The concentration distribution profiles of the MRI images were directly projected to the nodes in the finite element model as the initial condition for each time point. With this method, we can handle the situation that the concentration distribution profile was not symmetric.

### 2.4. Solution methodology

The concentration distribution in the vitreous was solved using the finite element method involving linear algebraic equations of the form,

(3) $$[K_{C}]\left\{\frac{dC}{dt}\right\}+D[K_{D}]\{C\}=0,$$

where [*K_C_*] and [*K_D_*] are the global system matrices developed from the Galerkin finite element method in appendix A. The solution employed the 3-D mesh of a total of 105,276 elements, and four nodal tetrahedral elements were used in the 3-D continuum domain. For the time discretization, we applied the well-known theta-method, which results in the equation

(4) $$[K_{C}]\left\{\frac{C^{n+1}-C^{n}}{\Delta t}\right\}+D[K_{D}]\{\theta C^{n+1}+(1-\theta )C^{n}\}=0$$

here *C^n^* is the concentration distribution at a specific time point, *C^n^*^+1^ is the concentration distribution at the later time point, and the parameter *θ* is related to the applied numerical method. It is worth emphasizing that for *θ* = 0.5, the method yields the Crank-Nicolson implicit method which has higher accuracy and unconditional stable for the time discretization [9]. Substituting *θ* = 0.5 in equation (4), then it can be rewritten as,

(5) $$\left([K_{C}]+0.5\Delta tD[K_{D}])\{C^{n+1}\}=([K_{C}]-0.5\Delta tD[K_{D}])\{C^{n}\right\}$$

In this equation, the concentration profile *C^n^* was known by projecting the concentration values of a specific time point from MRI images onto the finite element domain. The unknown parameter, *D*, was floated in order to get the calculated *C^n^*^+1^ which represents the concentration at the later time step. The value of *D* between two consecutive time points can be obtained by least square fitting of *C^n^*^+1^ and the concentration profile of the later time point from MRI images.

### 2.5. Model validation

In order to verify the finite element diffusion model developed in the previous section, the solutions obtained from the model was compared with the exact solutions. The exact solution for a spherical bolus of radius *r_0_* in a spherical shell of radius *R* was derived in the previous study [8] and shown in equation (6).

(6) $$c(r,t)=c_{0}\;\left[\frac{r_{0}^{3}}{R^{3}}+2\sum_{n=1}^{\infty }\frac{\sin(\lambda_{n}r_{0})-\lambda_{n}r_{0}\text{cos}(\lambda_{n}r_{0})}{\lambda_{n}R\;\sin^{2}(\lambda_{n}R)}e^{-D\lambda_{n}^{2}t}\frac{\sin(\lambda_{n}r)}{\lambda_{n}r}\right]$$

where *c_0_* is the initial concentration of the drug deposited, *r* is the radial distance from the point of injection, *D* is the diffusion coefficient, *t* is the time, *c*(*r*, *t*) is the concentration, and *λ_n_* is the set of constants satisfying the transcendental equation.

(7) $$\lambda_{n}R=\tan(\lambda_{n}R)$$

**Figure 2:**
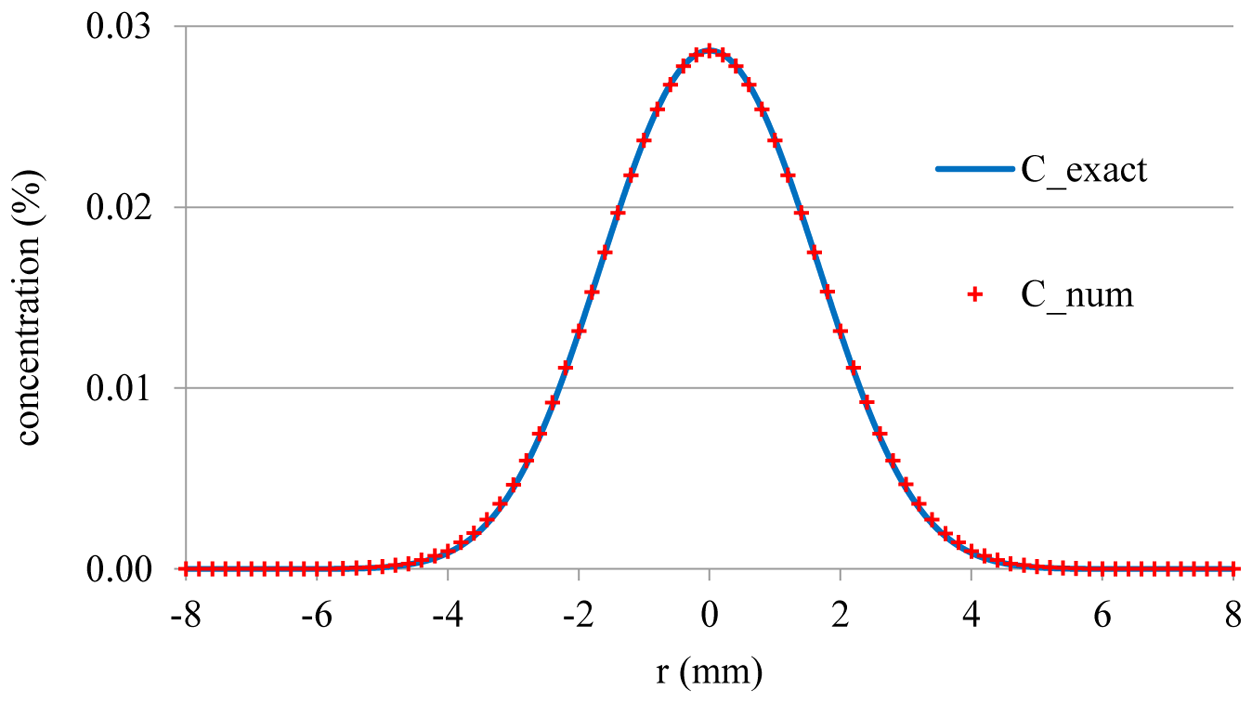
The comparison of concentration distribution profiles from the finite element model and the exact solution.

For the model validation, we use the following parameters: *r_0_* = 2 mm, *R* = 10 mm, and *c_0_* = 0.05 in both finite element model and the exact solution, to compare the concentration values. Figure 2 shows the comparison of concentration distribution profiles from the developed model and the exact solution under the same initial and boundary conditions. The finite element solutions along the center line at a specific time (t=40 min) have a strong agreement with the exact solutions.

## 3. Results

**Figure 3:**
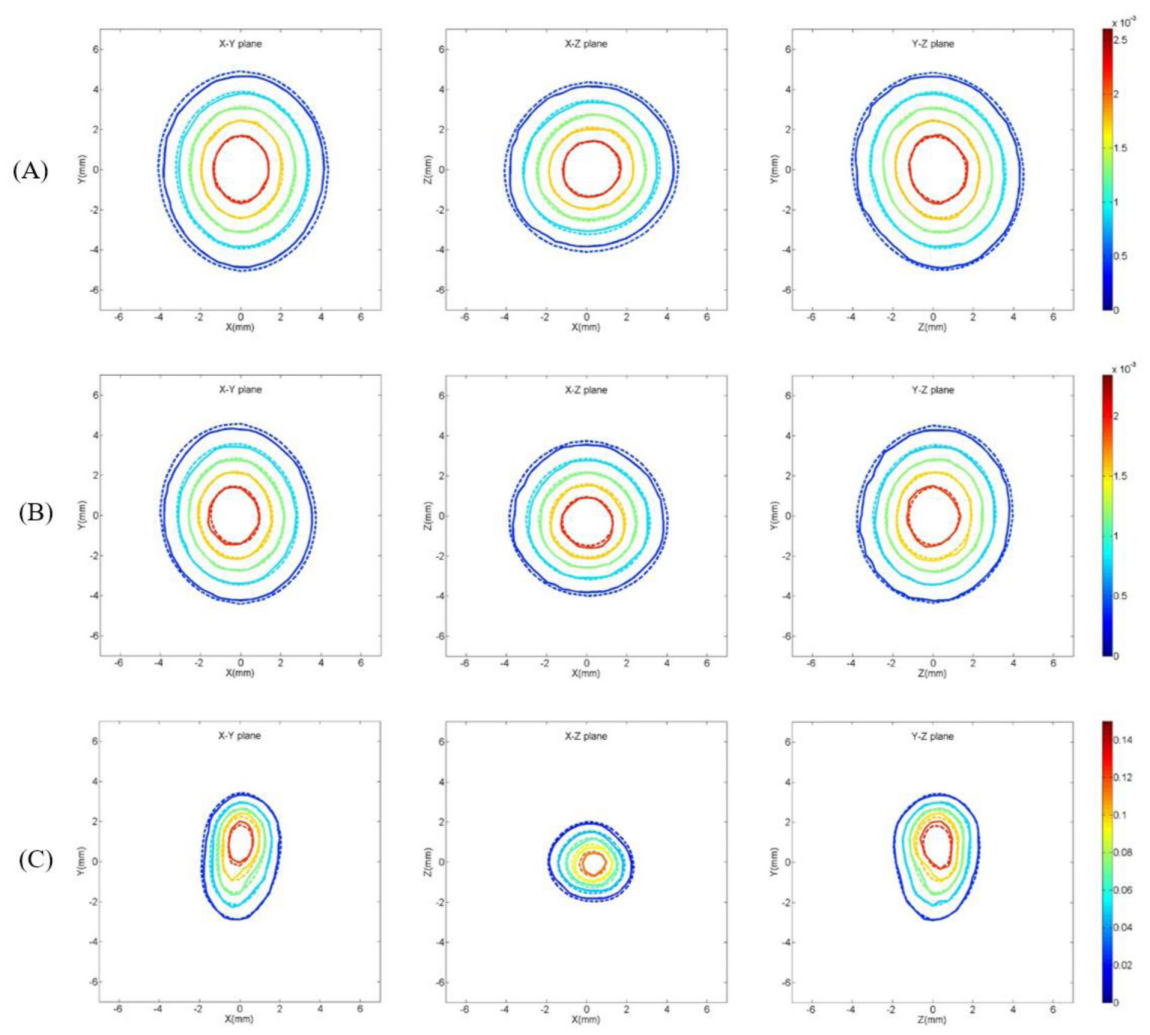
Contours of constant concentration in (A) Gd-DTPA at t=85 min, (B) Prohance at t=88 min, and (C) Galbumin at t=185 min (—: measurement, ----: finite element).

**Figure 4:**
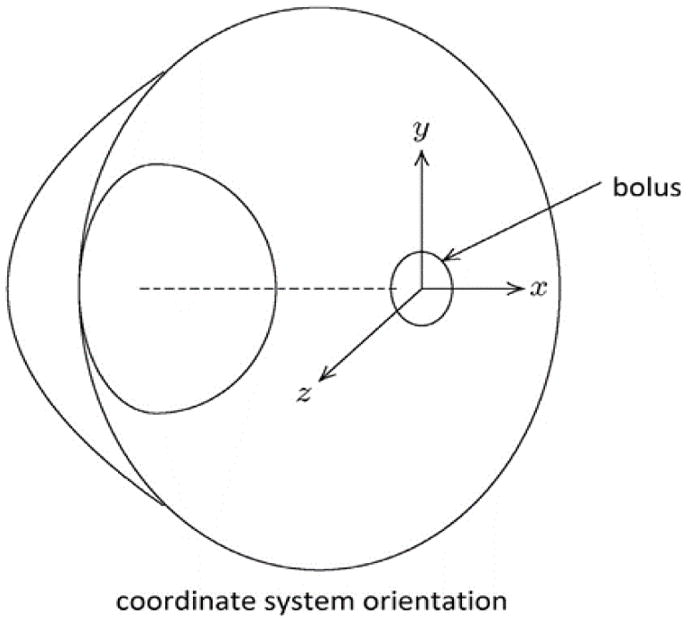
Coordinate system orientation with respect to the eye. The origin located at the center of the bolus.

**Figure 5:**
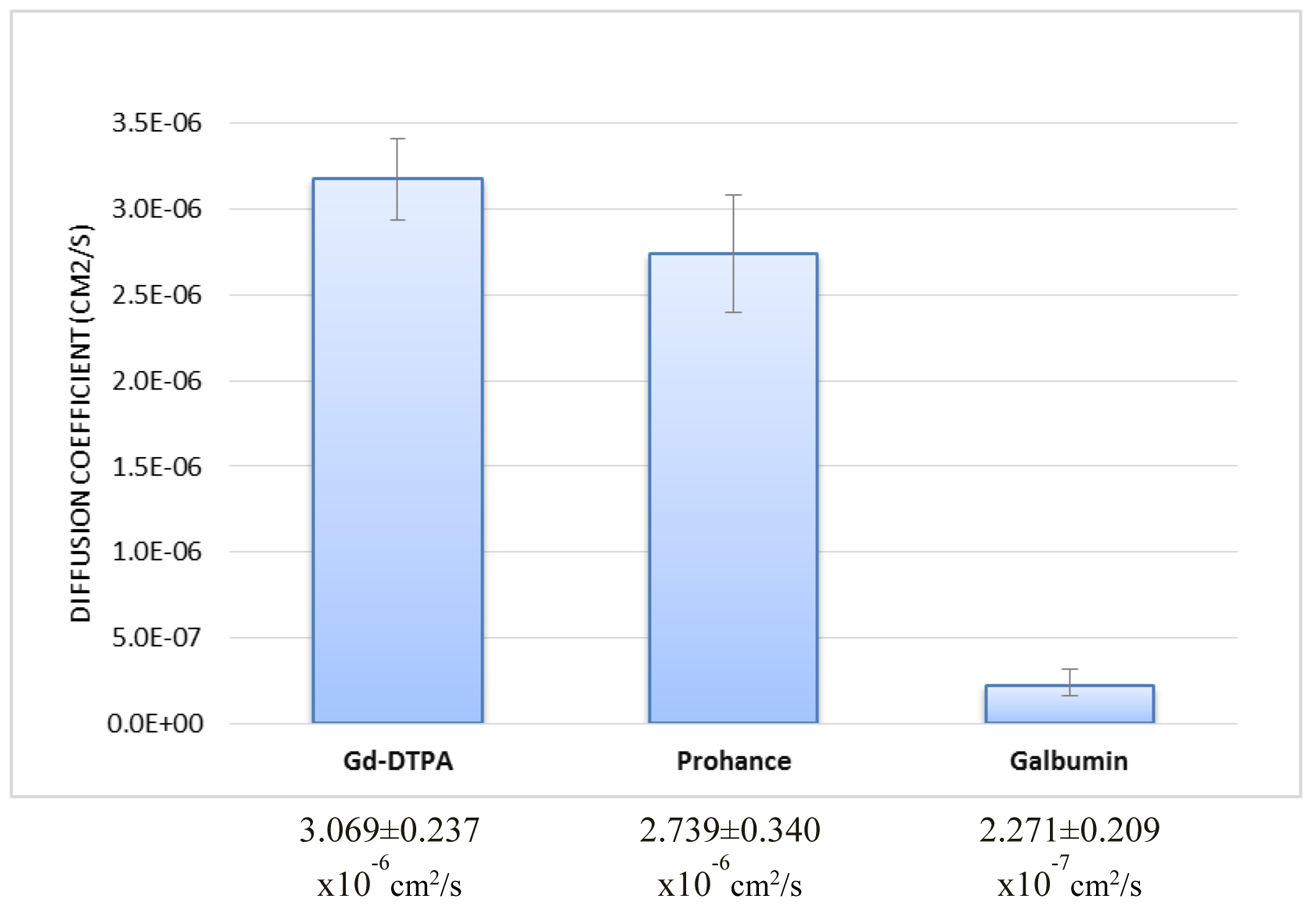
Diffusion coefficients of drug surrogates in bovine eyes.

Following the method of diffusion coefficient measurement by using MRI developed earlier, contours of constant concentration were established for different drug surrogates in each eye, and the comparison of these with the corresponding finite element calculation was shown in figure 3 where the (x, y, z) coordinate system in relation to the eye is shown in figure 4. While several time points for each eye were recorded and used in the analysis, only a selected example for each contrast agent is shown for illustrative purposes. The diffusion coefficients values of each drug surrogates acquired from the finite element method were presented in figure 5. We have made a comparison of previously reported D values for Gd-DTPA from the analytical method in the previous study [8] with the current method (finite element), and found that they were very consistent. The diffusion coefficient was (3.040 ± 0.274) ×10^−6^ cm^2^/s from the analytical method, and (3.069 ± 0.237) ×10^−6^ cm^2^/s from the finite element method. By comparing the diffusion coefficients between Gd-DTPA (molecular weight 938 Da), Prohance (molecular weight 559 Da), and Galbumin (molecular weight around 74k Da), we found that Galbumin had the lowest diffusivity, while Gd-DTPA had the highest diffusivity in the vitreous.

## 4. Discussion

Early investigations of diffusion coefficients of different MRI contrast agents had been conducted by Gillis et al [10]. They measured diffusivity of a negatively charged contrast agent Gd-DTPA and the non-ionic gadoteridol, Prohance, in control cartilages using MRI. The diffusion coefficients for Gd-DTPA and Prohance were reported by Gillis et al as (1.84 ± 0.12) ×10^−6^ cm^2^/s and (1.55 ± 0.22) ×10^−6^ cm^2^/s respectively. In our study, we found that the diffusion coefficient in the vitreous of a smaller molecule, Prohance was (2.739 ± 0.340) ×10^−6^ cm^2^/s which was lower than the diffusion coefficient of a larger molecule, Gd-DTPA. This implied that the effect of negative net charge on Gd-DTPA is likely to enhance the diffusivity in the vitreous.

Galbumin used in this experiment is a bovine albumin conjugated with gadolinium which has negative charge. Molokhia et al estimated the free aqueous diffusion coefficient of Galbumin in water at 37 °C as 8 ×10^−7^ cm^2^/s using the diffusion coefficient of bovine serum albumin (BSA), viscosity of water, and molecular weights of Galbumin and BSA [11]. The diffusion coefficient of Galbumin in this study was found to be (2.271 ± 0.209) ×10^−7^ cm^2^/s which is in the same order as the one estimated by Molokhia. Our lower value can also be attributed to the fact that we conducted experiments at room temperature. To compare with the smaller negative charge Gd-DTPA, Galbumin has a lower diffusion coefficient as expected.

## 5. Conclusion

The contour method recently developed is a very useful technique for the diffusion coefficient measurement in vitreous humor. With a new finite element model for the analysis, it can handle arbitrary shapes of the concentration contours, and we are able to show a comparison of diffusion coefficients of Gd-DTPA with Prohance and Galbumin. It has been found that both molecular weight and net charge of drugs affect the diffusion process in the vitreous humor. This method does not only work for the measurement in the vitreous humor, but also can be applied for other types of tissues in which the concentration profile can be visualized by MRI or fluorescence. The fact that we can now handle irregular shapes of the contours, the technique can be opened up to measure parameters that include convective transport effects as well.

## Figures and Tables

**Figure A1:**
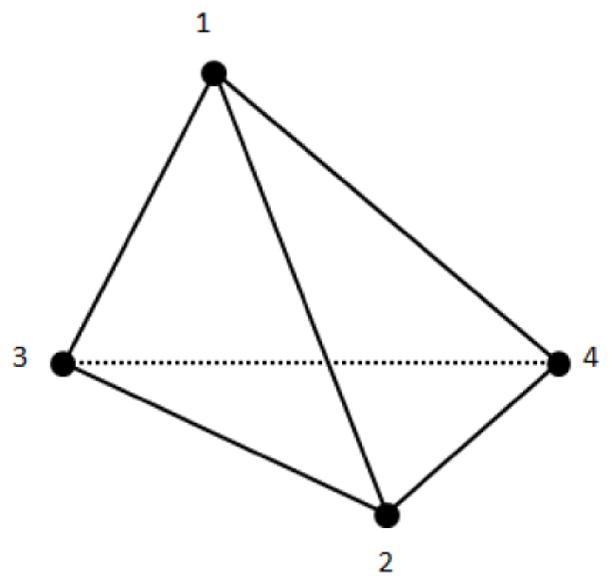
Linear tetrahedral element.

## Appendix A. Finite element equations for the intravitreal diffusion

This section provides a detailed derivation of ‘weak form’ of the drug diffusion equation using Galerkin method. Basic concept and application of this method can be found in literature [9]. The procedure starts by considering the suitable form of the mass transport equation which is given by equation (2),

(A1) $$\frac{\partial C}{\partial t}-D\;\left(\frac{\partial^{2}C}{\partial x^{2}}+\frac{\partial^{2}C}{\partial y^{2}}+\frac{\partial^{2}C}{\partial z^{2}}\right)=0$$

A domain V is divided into linear tetrahedral finite elements connected at nodes as shown in figure A1. Shape functions Ni are used for interpolation of concentration inside a finite element,

C=[N]{C}

where

$$[N]=[\begin{array}{llll} N_{1} & N_{2} & N_{3} & N_{4} \end{array}]\;\{C\}=\left\{\begin{array}{c} C_{1}\\ C_{2}\\ C_{3}\\ C_{4} \end{array}\right\}$$

Here {C} is a vector of concentrations at nodes and [N] is a matrix of shape functions. Using the Galerkin method, we can write equation (A1) in the following form:

(A2) $$\int_{V}N_{i}\;\left[\frac{\partial C}{\partial t}-D\;\left\{\frac{\partial }{\partial x}\left(\frac{\partial C}{\partial x}\right)+\frac{\partial }{\partial y}\left(\frac{\partial C}{\partial y}\right)+\frac{\partial }{\partial z}\left(\frac{\partial C}{\partial z}\right)\right\}\right]\;dV=0$$

Applying the divergence theorem to the terms in the bracket {}, we arrive at the relations:

(A3) $$\int_{V}N_{i}\frac{\partial C}{\partial t}dV+D\int_{V}\left[\frac{\partial N_{i}}{\partial x}\frac{\partial C}{\partial x}+\frac{\partial N_{i}}{\partial y}\frac{\partial C}{\partial y}+\frac{\partial N_{i}}{\partial z}\frac{\partial C}{\partial z}\right]\;dV-D\int_{S}\left[N_{i}\frac{\partial C}{\partial x}n_{x}+N_{i}\frac{\partial C}{\partial y}n_{y}+N_{i}\frac{\partial C}{\partial z}n_{z}\right]\;dS=0$$

where n_x_, n_y_, and n_z_ are outer normal components to the surface of the body. Since we applied the zero flux boundary condition at the surface of the body, the last terms in the integral of S is zero. Now the equation (A3) can be simplified as,

$$(\int_{V}\{N\}[N]dV)\{\frac{dC}{dt}\}+D\;\left(\int_{V}\left\{\frac{\partial N}{\partial x}\right\}[\frac{\partial N}{\partial x}]\;dV+\int_{V}\left\{\frac{\partial N}{\partial y}\right\}[\frac{\partial N}{\partial y}]\;dV+\int_{V}\left\{\frac{\partial N}{\partial z}\right\}[\frac{\partial N}{\partial z}]\;dV\right)\;\{C\}=0$$

Let’s denote

$$[K_{C}]=\int_{V}\{N\}[N]dV\;[K_{D}]=\int_{V}\left\{\frac{\partial N}{\partial x}\right\}[\frac{\partial N}{\partial x}]\;dV+\int_{V}\left\{\frac{\partial N}{\partial y}\right\}[\frac{\partial N}{\partial y}]\;dV+\int_{V}\left\{\frac{\partial N}{\partial z}\right\}[\frac{\partial N}{\partial z}]\;dV$$

The discretized finite element equation for the mass transport equation can be rewritten as in equation (3),

(A4) $$[K_{C}]\left\{\frac{dC}{dt}\right\}+D[K_{D}]\{C\}=0$$

## References

[1] Pflugfelder SC, Hernandez E, Fliesler SJ, Alvarez J, Pflugfelder ME, Forster RK (1987). Arch Ophthalmol.

[2] Ozkiris A, Erkilic K (2005). Can J Ophthalmol.

[3] Bhavsar A, Ip M, Glassman A (2007). Am J Ophthalmol.

[4] Stay M, Xu J, Randolph T, Barocas V (2003). Pharm Res.

[5] Balachandran R, Barocas V Pharma Res.

[6] Kathawate J, Acharya S (2008). Int J Heat Mass Transfer.

[7] Haghjou N, Abdekhodaie MJ, Cheng YL, Saadatmand M (2011). World Acad Sci Eng Technol.

[8] Penkova A, Rattanakijsuntorn K, Sadhal SS, Tang Y, Moats R, Hughes PM (2014). Int J Heat Mass Transfer.

[9] Lewis RW, Nithiarasu P, Seetharamu KN (2004). Fundamentals of the Finite Element Method for Heat and Fluid Flow.

[10] Gillis A, Gray M, Burstein D (2002). Magnetic Resonance in Medicine.

[11] Molokhia SA, Jeong EK, Higuchi WE, Li SK (2009). Exp Eye Res.

